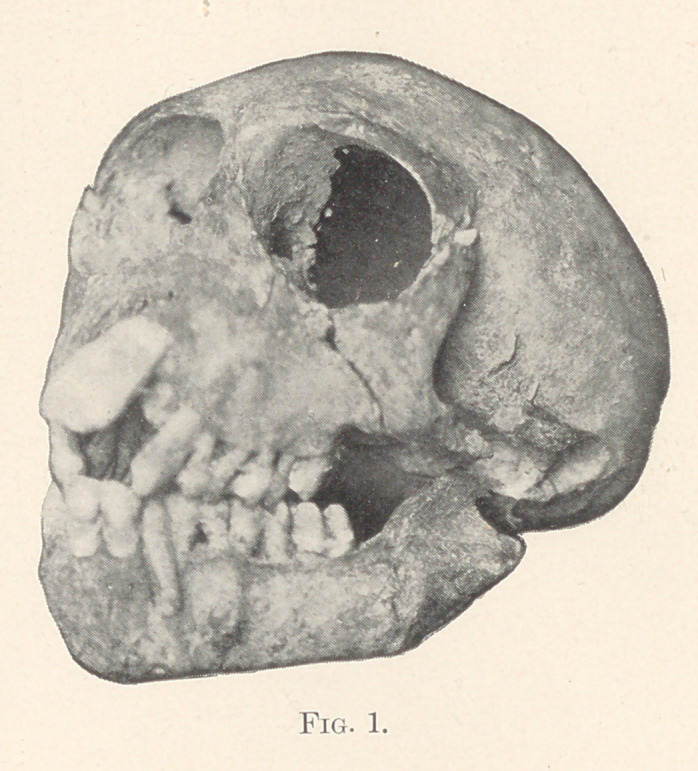# Interstitial Gingivitis Due to Autointoxication

**Published:** 1900-02

**Authors:** Eugene S. Talbot


### International Dental Journal.

Vol. XXI. February, 1900. No. 2.

### Original Communications.[1]

### INTERSTITIAL GINGIVITIS DUE TO AUTOINTOXICATION.

BY EUGENE S. TALBOT, M.D., D.D.S.[2]

In my work upon interstitial gingivitis, or so-called pyorrhœa alveolaris, I, of necessity, divided the causes into local and constitutional. Conclusions and treatment were based entirely upon pathology and not upon previous ideas or methods of treatment. Exceptions have been taken to these conclusions and treatment by some of the best men in the profession.

Every one will agree that successfully to treat a disease the cause should be removed. There are many able practitioners who do not believe in the constitutional nature of interstitial gingivitis. A majority of the profession do not believe that constitutional treatment is necessary to successful issue. It is with a view of making this position more clear that the present paper is written.

I propose to direct attention to the constitutional variety of the disease,—interstitial gingivitis of the type due to autointoxication.

Every dentist even in practice but a short time has noticed that alveolar processes and gums recede from the necks of the teeth through the entire dental arch, or, perchance, but one or two teeth

[1] The editor and publishers are not responsible for the views of authors of papers published in this department, nor for any claim to novelty, or otherwise, that may be made by them. No papers will be received for this department that have appeared in any other journal published in the country.

[2] Fellow of the Chicago Academy of Medicine.

so involved at different localities in the mouth. The alveolar process is hard, and the gums, as a rule, are healthy, although there may be occasionally a low form of gingivitis and exceptionally a discharge of pus about the necks of the teeth. Despite the infrequency of the pus discharged, the condition has been styled "pyorrhœa alveolaris." The patients in whom this condition may be observed are often seemingly healthy. The jaws are well developed, and the number of teeth normal, with broad and short crowns well set in the jaw of the type seemingly least prone to decay. The general appearance of the patient suggests robust health. This condition, while not confined to one sex, most frequently occurs in the male. It is not confined to any period of life, but may occur at any time after puberty.

In dealing with the etiology of this disease, the tissues involved and their physiology first require attention, since here is to be often found the explanation of predisposition to pathologic change. Three great factors require attention. In the first place, in the evolution of the face, the jaws have received receding tendencies, antero-posteriorly and laterally. They are much smaller to-day than formerly, and are still decreasing in size. The crowns of the teeth are not so large and the spaces between the roots are gradually diminishing, hence less alveolar process is required. In the second place, it must be remembered that the existence of the alveolar process depends upon the existence of the teeth. When the teeth are lost the processes disappear by absorption. In the third place, senile absorption occurs just as in the bones of the body, but to more marked extent. In the mouth and jaws, therefore, transitory or adventitious structures occur, which are more predisposed to disease than permanent structures. This is why the alveolar process is more subject to changes produced by altered metabolism due to trophic derangement of nutrition than other structures. Hence osteomalacia, or senile absorption, occurs with more rapidity and disastrousness in the alveolar process than in other bones of the body. In other words, a cause which would not influence bone absorption elsewhere would markedly affect the alveolar process.

This process, furthermore, being situated in a cavity which plays an excretory part in an exceedingly moderate degree under normal conditions, would be markedly affected by the strain and influences resultant on excessive strain on other excretory organs. Such a strain would occur from the old condition known as "blood impurity," and for which blood purifiers were taken in the spring ere the days when vegetable food and fresh meat were accessible to the general population. This condition, which was scientifically referred years ago to "rheums" or "humors" (whence the name rheumatism), is now known as autointoxication. This condition, while manifesting itself under different phases as the so-called gouty or uric acid states or rheumatism, is at the bottom, and, so far as clinical phenomena and treatment are concerned, but one process. Under existing data the position taken by Rhein[1] and others as to the different clinical aspects of autointoxication cannot be maintained. According to recent investigations by Albu, the autointoxications may be divided into the following classes: Autointoxications from the suppression or disturbance of the functions of an organ,—*i.e.*, autointoxication of thyroid gland, pancreas, liver, suprarenal capsules, producing myxœdema, diabetes, acute yellow atrophy, and Addison's disease; autointoxications which occur from anomalies in general metabolism without definite localization, such as rheumatism, gout, and oxaluria; autointoxications which are caused by the retention of the physiologic products of metabolism in different organs, such as poisoning due to extensive destruction of the skin by burning, carbolic acid poisoning, uræmia, and eclampsia; autointoxication due to the over-production of physiologic and pathologic products of the organism, such as ammonemia, acetonuria, diaceturia, diabetic coma, etc. The most frequent source of this intoxication is the gastro-intestinal tract.

Autointoxication, like all intoxication, comprehends, as W. A. Evans has said:[2] (1) production of the intoxicant; (2) absorption thereof; (3) reaction thereto. These three are embraced when autointoxication is spoken of, which is poisoning of an organism with matter produced by itself. Assimilation or the making of tissue is the passing of the simple into the complex, stability into instability, with the storing of energy. This instability is a necessity of life. Dissimilation, divided into two divisions, death and energy, the last being a modification of death, is the passing of the complex to the simple, the instable to the stable, with the liberation of energy.

In the building-up process the unused portions of the absorbed foods may produce autointoxication. In the breaking-down process the ash can produce autointoxication. So long as these two processes —tissue building and tissue waste—are normal, intoxication can

[1] The value of Rhein's researches, however, as to the general principle of autointoxication cannot be too much estimated.

[2] Journal of the American Medical Association, vol. xxix.

only ensue from faulty action of the destroying organs, of which the liver is the chief, or of the eliminating organ, of which the kidney is a type. This constitutes the first group of those due to faulty elimination. It applies to food remnants and to tissue waste, both normal and pathologic.

The second group is due to errors in cell life. It occurs under three sub-types: (1) by some reason food elements are left unused; (2) the ash from food-burning is usually toxic or unusually difficult to absorb; (3) the secretion of the cells is toxic.

While it may be stated, in accordance with the principles just laid down, that, considered from the direct stand-point of the production in the body, there can be no bacteriology of autointoxication, still it must be admitted that autointoxication produces culture mediums in the body which would not otherwise exist, which enhance the virulency of the microbe, and hence increase the toxicity of its ptomaine. Indirectly, therefore, autointoxication must be considered a factor in bacterial action. In dealing with the general question of autointoxication, it should be remembered that when proteids are placed under the action of gastric and pancreatic juice, they are changed into a hemi- and an anti- group. The anti- group is, as J. A. Wesener points out, broken down into antialbumose and a small quantity of antipeptone. This last is a stable body which does not yield to the digestive juices or even to dilute sulphuric and hydrochloric acids. It is absorbed by the small intestine, but does not replace any waste of the used-up proteids of the body. Antialbumose is changed to serum albumin, and is the one that furnished the body with its proteid food. The albumose, when injected subcutaneously, causes death; the blood fails to coagulate by reason of the fact that the lime-salts are precipitated by this body. If for any reason the epithelium of the intestine fails to perform its functions of changing this body into serum albumin, toxic symptoms will arise.

While uric acid is charged with being the chief factor in autointoxication, its importance has been over-estimated. It is a thermometer of the extent of autointoxication rather than its chief factor. Recent investigations,[1] as J. A. Wesener remarks, show that uric acid represents the metabolism of the nucleins of the body, and is in no way related to the albumins taken in as a food, for these last bodies of this group are very poisonous. The necrosis arises as follows: The leucoctyes break down easily because the

---

[1] Journal of the American Medical Association, vol. xxix.

carbodioxide remains too long in the tissue spaces; the nucleinic acid which is liberated in this way attacks the connective tissue, etc., irritates it, and this forms a good basis for destruction of nucleins.

A factor in autointoxication is non-performance of the process of elimination by the various excretory organs. In the urine alone, as Bouchard has shown, there is present each day sufficient toxins in a normal individual to cause death if not excreted. This condition is notoriously increased after prolonged nervous explosions like those of epilepsy or hysteria. This was pointed out thirty years ago by Meynert, who showed that the status epilepticus, or condition of repeated convulsion, was due to the accumulation of a proteid or nitrogenous body in the system. This status epilepticus is preceded by a decreased amount of toxin in the urine and succeeded by an increased amount. The same is true as to the influence of non-elimination from the excretory organs (the bowels, lungs, and oral cavity) as well as the non-exercise of its poison-destroying powers by the liver. The non-elimination factor, moreover, interferes with ordinary digestive functions, and hence increases its own extent. The other factor in autointoxication is the production of toxic products in such quantity as to prevent their destructions by organs like the liver and consequent elimination, since a product to be properly eliminated must be reduced to a chemical type. Among the factors which tend to produce both these elements of elimination is the power exercised over the processes of growth and repair by the nervous system. In part this influence is exerted through the control of blood allowance by the vasomotor nervous system, and in part by the direct control of the nervous system over tissue change, which is known as its trophic function. Both these influences are effected by mental and nerve strain.

As Bichat showed decades ago, sudden emotion may produce marked effects upon the secretion of bile, and occasion jaundice. Cases are far from infrequent where emotions like jealousy may produce a mimicry of gall-stone colic in neuropathic individuals. Murchison, Christison, and Thompson have traced attacks of biliary colic to jealousy. Other liver changes from sudden nervous disturbances, whether of a mental type or not, are not rare. As mental impressions are communicated to the central nervous system purely through mechanical changes in the nerves, such influence must be purely material in operation. As the brain exercises a checking influence on the operations of the liver it is obvious that these mental influences can produce two effects. First, the mental shock might increase the checking action of the central nervous system on the local ganglia of the liver. Second, the mental shock might destroy the checking action on the liver ganglia, and in consequence the liver ganglia go too fast, resulting in their exhaustion. Either of these two conditions would interfere with a poison-destroying action of the liver, and accumulation of waste product would be the result. What is true of the liver is true of the other organs. This is especially noticeable, as Tuke points out, in regard to the kidneys. The action of mental anxiety or suspense in causing a copious discharge of pale fluid is familiar enough to all, especially to the medical student about to present himself for examination, the amount being in a pretty direct ratio to his fear of being plucked. The frequency of micturition may, however, arise from nervous irritability of the bladder without increase or even with diminished secretion. Still the action of the skin is usually checked, the extremities are cold, and the kidneys have to pump off the extra amount of fluid retained in the circulation. There is not elimination of the substances usually separated from the blood, compared, at least, with the aqueous character of the whole secretion. The odor may be effected by the emotions in man as in animals. Prout is of the opinion that mental anxiety will not only produce non-elimination, but also change in the chemical character as indicated by the odor and otherwise. As Claude Bernard long ago showed, disturbances in the medulla produce a markedly pale excessive urine. These disturbances often arise from intellectual strain or emotional shock. The influence of emotional states on secreting processes, and thereby indirectly upon autointoxication states, is illustrated in the fact long ago pointed out by Tuke, that pleasurable emotions increase the amount of gastric juice secreted, the opposite effects being produced by depressing passions. Beaumont found in a man with a fistulous opening in the stomach that anger or other severe emotions would cause its inner or mucous coat to become morbidly red, dry, and irritable, occasioning at the same time a temporary fit of indigestion.

The influence of fear and anxiety on the bowels is as well marked as that upon the bladder and kidneys. Apart from muscular action, defecation may become urgent or occur involuntarily from various causes; the increased secretion from the intestinal canal, as from fear, and in some cases from the altered character of the secretion itself. While in this respect the influence of fear may be inconvenient in man, it naturally assists escape in some animals, as the polecat.

The emotions powerfully excite, modify, or altogether suspend, as Tuke has shown, the organic functions. This influence is transmitted not only through the vasomotor nerves, but through nerves in close relation to nutrition and secretion. As when the excitement is of peripheral origin, a sensory or afferent nerve excites their function by reflex action, so that when emotion arises it may excite the central nuclei of such afferent nerve and this stimulus be reflected upon the efferent nerve, or it may act directly through the latter.

The pleasurable emotions tend to excite the processes of nutrition, hence the excitement of certain feelings may, if definitely directed, restore healthy action to an affected part.

Violent emotions modify nutrition. Various forms of disease originating in perverted or defective nutrition may be caused primarily by emotional disturbance.

As respects secretion, the emotions, by causing a larger amount of blood to be transmitted to a gland, increase sensibility and warmth, and stimulate its function or directly excite the process by their influence on nerves supplying the glands. Painful emotions may modify the quality (*i.e.*, the relative proportion of the constituents) of the secretions.

Emotions check secretions either by extreme acceleration of blood through a gland by unduly lessening its afflux or by direct influence upon the gland. Although, as a rule, the activity of those glands which bear special relation to an emotion is in a direct ratio to its force, the secretion is checked when the emotion is excessive.

The pleasurable emotions tend to act only in one direction, that of increased activity of the secretions. The painful emotions act both in stimulating and arresting secretion. Thus grief excites the lachrymal and rage the salivary glands. Excess of grief checks the lachrymal and fear the salivary glands, while anxiety suspends the gastric. Extreme fear induces perspiration; fear causing less vascularity and secretion, the secretion of milk is lessened by it. The temperature of the skin is lowered and its secretion checked, although cold sweats may occur. Salivary secretion is arrested, while intestinal secretion is often increased.

The main immediate causes of autointoxication, aside from the factor which sets them in action or predisposes, are, according to Pocheore, diminished alkalinity of the blood, due to acidity of the tissues from over-exertion and other causes. Insufficient supply of oxygen, abnormal fermentation process in the intestines, poison from without by bacterial or other agencies, retention of metabolic processes, evidences of these conditions, may often be detected in the urine. The resisting power of the organism depends very largely upon the manner in which the internal or tissue respiration is carried on.

The four great sewers which eliminate the waste products of the body are the kidneys, the skin, the lungs, and the bowels. Shut off one of these floodgates, and not only the others but even secretory organs must do the work of the one disabled.

One of the greatest eliminators of effete matter is the skin. If from any cause the skin becomes diseased, or after or during eruptive fevers in children, trophic changes and autointoxication are very marked. Pits and grooves upon the teeth, loss of hair, and nail diseases are familiar to every practitioner. Fortunately, these diseases have little or no permanent effect upon the gums, because they are yet in the constructive stages. It is only after one has obtained his growth that autointoxication from the skin lesion, or, indeed, from any cause, makes a grave and marked impression upon the alveolar process. This is peculiarly noticeable in animals. When the other excretory organs are acting badly, the skin and mouth assume their functions. Under massage the skin of the self-poisoned patients has a fecal odor, even when the bowels and kidneys are acting apparently well.

Autointoxication due to imperfect elimination of effete matter from the lungs is a fruitful source of interstitial gingivitis. The more marked forms are those of tuberculosis, in which there is great debility and in which there is greater waste than repair. Self-poison is continually going on and will continue until death. The chest capacity for the inhalation of pure air is almost *nil*, hence the blood is improperly oxygenated and it soon ceases to convey nutriment to the tissues. Eighty per cent. of criminals who die of tuberculosis in prisons have undeveloped chest walls. The degeneracy, therefore, cuts quite a figure in the *rôle* of autointoxication. Degenerates with contracted chest walls are, however, more frequently found. Many undeveloped individuals in every walk of life have tuberculosis. People with undeveloped chest walls and chest capacity may not have tuberculosis and yet suffer from autointoxication. Those who have had pneumonia with adhesion, and who are thus unable to oxygenate the blood, are subject to this disease.

Asthmatics and hay-fever patients suffer from autointoxication and alveolar absorption. When the skin is overstrained, as to excretion through kidney and bowel overstrain, the lungs are forced to take on increased work with imperfect oxygenation as a result. This is noticed in the odor of the breath in Bright's disease. In nerve-strain states, and in the conditions described by Albu, not only do the excretory organs suffer, but the secretions, like those of the salivary and other buccal glands, are so altered as to become irritants. These excretory conditions result not only upon autointoxication states, but are modified trophic nerve function alteration.

By trophic changes is meant such tissue alterations as occur in morbid conditions from disordered function of the centres controlling nutrition. There may be peripheral as well as central tissues involved. The well-known law of Wallerian degeneration of nerve-fibres is an illustration: the posterior spinal ganglion acting as a trophic centre for the fibres of the posterior root is in the cord itself. The trophic action may be therefore peripheral, though, as a rule, in extensive changes central (cerebral or spinal) origin should be looked for.

The more marked instances of trophic disturbances are the wasting of limbs in the spinal paralysis of children or adults, and the most striking are perhaps the cases of progressive muscular atrophy of the various types and the peculiar hemiatrophy or hypertrophy of the face and other parts. Besides atrophy or hypertrophy there are included under this head other changes in structure or growth, such as the necrosis of some "bedsores," those of the hair or nails, etc. These often indicate a general systemic disturbance, but may be more or less local in their origin likewise. In the case of the bedsores the condition is a necrosis due to the mildest kind of traumatism, a simple pressure acting on tissues, the nutrition of which is in a thoroughly depraved condition, and which consequently break down under the slightest provocation. The direct cause is external, but the primary condition is a general one, starting from the great centres, controlling growth and repair of the system.

Trophic changes are not always destructive; they may be the reverse, as already indicated. A general tendency to take on fat may be considered in a wider sense of term to be a trophic affection, but the term is usually limited to the more or less atrophies or hypertrophies,[1] the changes in structure, pigmentation, etc., which

[1] Evolution by Atrophy, Demoor, 1899.

follow certain nervous conditions and are spoken of as being under the influence of a special class of nerves, the trophic nerves, which are supposed to govern nutrition. These are known physiologically rather than anatomically; they have not been isolated, and their independent existence is still somewhat in question.

A trophic change allied to that of interstitial gingivitis is the change which takes place in the skin at the finger-tips in the fall of the year. Through the hot weather the effete matter has been carried off through the perspiration, when cool weather returns the skin ceases to act, and the liver, kidneys, and bowels must do the work of the skin. Autointoxication results, nutrition is cut off, and the skin of the fingers peels off, leaving the basement membrane layers exposed and tender. The exfoliation of the skin continues until the system has adjusted itself to the new order of things.

As I have elsewhere pointed out, the great neuroses, like locomotor ataxia and paretic dementia, afford instances of trophic disorders directly underlying this factor in interstitial gingivitis. This was lately brought anew to the profession by M. Raoul Beaudet, under the name of "mal perforant buccal" ("Thesis of Paris," 1898). This malady, of an evolution more or less rapid, is essentially marked by the shaking and falling out of the teeth, by alveolar resorption, and gingival ulceration, by the perforation, and at times necrosis of the maxillary. M. Beaudet reports seven cases of perforation, three of which came under his personal knowledge. Since then M. Letulle has published (*Presse Médicale* of April 2, 1898) a new case. Dr. Chagnon reports the following case:[1]

O. G. is forty-four years of age. About ten years ago he contracted syphilis, for which he was treated more or less regularly. Two years later he got married and had healthy children. He did not abuse himself by the use of alcoholic liquors. In June, 1895, he was admitted to St. Jean de Dieu Asylum, suffering from intense maniacal excitement. At the end of two months the excitement disappeared and the physical and psychical symptoms of paretic dementia, until then hidden by his state of excitement, commenced clearly to show themselves,—embarrassment of speech, fibrillar twitchings of the tongue, ideas of greatness and wealth, and, to crown all, a state of dementia.

The disease followed its course without any remarkable incidents until near September, 1897. At this date my attention was called

---

[1] The American Journal of Insanity, October, 1899.

FIG. 2.—Interstitial gingivitis resulting in osteomalacia, due to autointoxication. Old dog. *A*, alveolar process ; *B*, large spaces arising from absorption of trabeculæ starting in the Haversian canals ; *C*, perforating canal absorption ; *D*, halisteresis ossium, or decalcified bone ; *E*, arteries originally Haversian canals.

FIG. 1.

to the state of his dental system. On examination the two incisors, the canine, the premolars, and the first molar of the left upper maxillary were found to be very loose and had only to be picked out. All the teeth were absolutely sound. The ulceration following the loss of the teeth, and which affected the surface of the alveoli, did not heal. About the middle of September a sequestrum became detached. As you can see in this, the work of alveolar resorption is not yet much advanced. The palate roof, forming the anterior border of the maxillary sinus, also forms part of the sequestrum, and thus there was a large aperture of communication between the sinus and the buccal cavity. Two months later the ulceration was cicatrized.

*Present condition.*—In the inferior jaw all the teeth are sound and there are none wanting. The two premolars and the right canine of the upper jaw are decayed, the second and third left molars, as well as the first right molar are loose, but perfectly sound. There exists no alveolar pyorrhœa, neither does any trace of ulceration appear, except a small opening which would not admit the probe.

It was impossible to inquire into his sensibility owing to the profound state of dementia, which rendered him incapable of understanding the questions put to him. His physical condition is yet good; he is only troubled with weakness of the limbs. The rapid evolution of the affection is noticeable: less than two months after the falling out of the teeth the sequestrum became detached. This would explain the rather slow degree of alveolar resorption. The disease continues its course, since the second and third left molars and the first right molar are actually loose.

Many cases of this type could be cited, but this one so represents the clinical phenomena as to make their report a vain repetition.

It will be observed in a general way that disease of any great eliminator of effete matter from the body, the lungs, skin, kidneys, and bowels, or any disease of the body which may interfere with the function of waste and repair, will produce autointoxication. Autointoxication produces irritation, inflammation, and absorption of the alveolar process. The constitutional variety of interstitial gingivitis is a definite result of trophic and metabolic change in the system. These changes, if not corrected, lead to severe results. These gum disturbances frequently prophesy the future course of autointoxication of which they are the initial symptom.

Many illustrations could be produced to demonstrate interstitial gingivitis due to autointoxication from casts in my possession, representing all diseases, but the following figure is a more forcible illustration than any that could be produced. It is a skull of a monkey, about one year old. He died of tuberculosis. All the temporary teeth except the left central incisor are in place. Absorption of the alveolar process has taken place to such an extent that one tooth (the central incisor) has dropped out. The inferior and superior left cuspids are ready to drop out, and all the other teeth could be removed with the fingers. Fig. 2 is the microscopic specimen of osteomalacia or senile absorption of the jaw of an old dog.

## Figures and Tables

**Fig. 2. f1:**
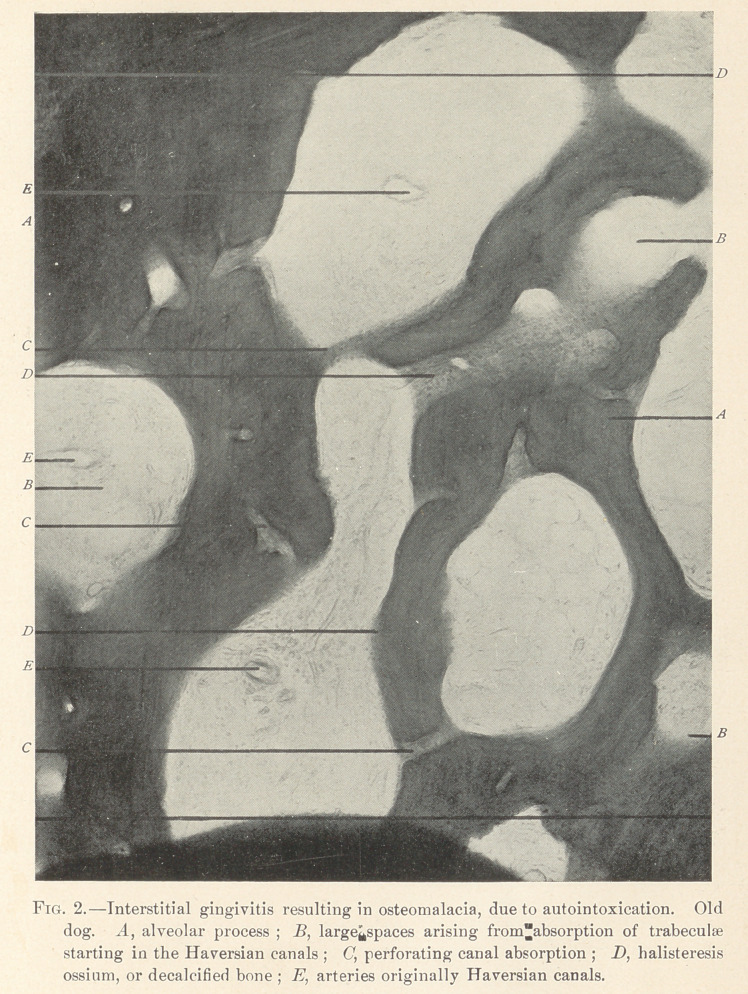


**Fig. 1. f2:**